# Association of Interleukin-6 Signaling and C-Reactive Protein With Intracranial Aneurysm: A Mendelian Randomization and Genetic Correlation Study

**DOI:** 10.3389/fgene.2021.679363

**Published:** 2021-06-08

**Authors:** Peng-Peng Niu, Xue Wang, Yu-Ming Xu

**Affiliations:** Department of Neurology, The First Affiliated Hospital of Zhengzhou University, Zhengzhou, China

**Keywords:** C-reactive protein, inflammation, interleukin-6, intracranial aneurysm, Mendelian randomization

## Abstract

**Background and objective:**

Evidence suggests that interleukin-6 (IL6) signaling is causally associated with aortic aneurysm independently of the effect of C-reactive protein (CRP). We aimed to explore the genetic overlap and associations between inflammation (IL6 signaling and CRP) and intracranial aneurysm (IA) risk.

**Methods:**

Two-sample Mendelian randomization (MR) methods were used to assess the causal effects of soluble IL6 receptor (sIL6R) (*n* = 21,758) and CRP (*n* = 204,402) levels on IA (7,495 cases and 71,934 controls) risk using genome-wide association study summary data of European individuals. Cross-trait linkage disequilibrium score regression was used to estimate the genetic correlations of CRP (*n* = 400,094) with IA.

**Results:**

MR analyses showed that circulating sIL6R and CRP levels were not associated with the risk of IA. The odds ratios based on the inverse variance-weighted method were 0.986 (0.950–1.023, *p* = 0.45) and 0.957 (0.846–1.084, *p* = 0.49) for sIL6R and CRP, respectively. MR analyses using data of ruptured and unruptured IA each showed no association. Linkage disequilibrium score regression showed that the genetic correlation between CRP and IA was 0.16 (SE = 0.04, *p* = 0.0003). The genetic correlation diminished after conditioning IA on blood pressure (0.07 ± 0.05, *p* = 0.16), smoking (0.02 ± 0.05, *p* = 0.65), or blood pressure plus smoking (−0.03 ± 0.05, *p* = 0.53).

**Conclusion:**

Using associated genetic variants as instrument variables, two-sample MR analyses showed no evidence that circulating sIL6R and CRP levels were associated with IA risk. Although a positive genetic correlation was found between CRP levels and IA risk, it was mainly driven by the shared genetic background of blood pressure and smoking with both CRP and IA.

## Introduction

Intracranial aneurysm (IA) is characterized by balloon-shaped dilatation of the vascular wall of intracranial arteries, occurring predominantly in the circle of Willis. The prevalence of unruptured IA is 3.2% (Vlak et al., 2011). Rupture of IA can cause subarachnoid hemorrhage (SAH), which is a severe type of stroke with high fatality and morbidity rates (Xu et al., 2019). The annual incidence of aneurysmal SAH ranges from 1 per 100,000 persons to 27 per 100,000 persons (Dong et al., 2019), worldwide, with almost 500,000 individuals suffering from aneurysmal SAH each year (Hughes et al., 2018).

The primary risk factors for IA include positive family history, female sex, smoking, hypertension, and alcohol consumption (Vlak et al., 2013; Xu et al., 2019; Vyas et al., 2021). In addition, emerging evidence increasingly suggests that inflammation is a critical component in the pathogenesis of the development and rupture of IA (Penn et al., 2014; Signorelli et al., 2018; Furukawa et al., 2020). Various factors that regulate the inflammatory response are associated with IA pathogenesis, including leukocytes, complement, immunoglobulins, cytokines, and other humoral mediators (Chalouhi et al., 2012). As an important pro-inflammatory cytokine, and it has been suggested that interleukin-6 (IL6) may also be involved in the pathogenesis of IA (Gao et al., 2020). A meta-analysis showed that two IL6 promoter polymorphisms (−174G/C and −572G/C) were associated with IA (Zheng et al., 2013). However, another recently published meta-analysis failed to confirm this association (Hu et al., 2020). Furthermore, a case-control study showed that levels of C-reactive protein (CRP), which is a well-established downstream molecule of IL6 signaling, were higher in patients with fusiform IA than controls (Mota Telles et al., 2020). With the increasing availability of summary data from genome-wide association studies (GWASs), new methods such as two-sample Mendelian randomization (MR) and linkage disequilibrium score (LDSC) regression have been widely used to determine the relationship between traits. In two-sample MR analysis, single nucleotide polymorphisms (SNPs) associated with the trait of interest (e.g., IL6) from one GWAS are used as instrumental variables. Using the estimates of these SNPs for both exposure of interest (e.g., IL6) and outcome of interest (e.g., IA) from two separate GWASs, a two-sample MR study can assess the relationship between two traits. Although certain assumptions need to be met, two major advantages of a two-sample MR study are that it can assess causal relationships and eliminate the bias caused by conventional confounders.

Because the plasma soluble IL6 receptor (sIL6R) acts as a decoy receptor of IL6 signaling and its plasma levels are highly hereditary, sIL6R has been chosen as an indirect index of IL6 signaling in previous MR studies (Rosa et al., 2019; Kappelmann et al., 2021). Previous studies using the two-sample MR method have confirmed the causal effect of IL6 signaling on a range of cardiovascular phenotypes including stroke, coronary heart disease, and aortic aneurysm (AA) (Rosa et al., 2019; Georgakis et al., 2020). In addition, the effect of IL6 signaling was found to be independent of the effect of CRP (Georgakis et al., 2020).

**FIGURE 1:**
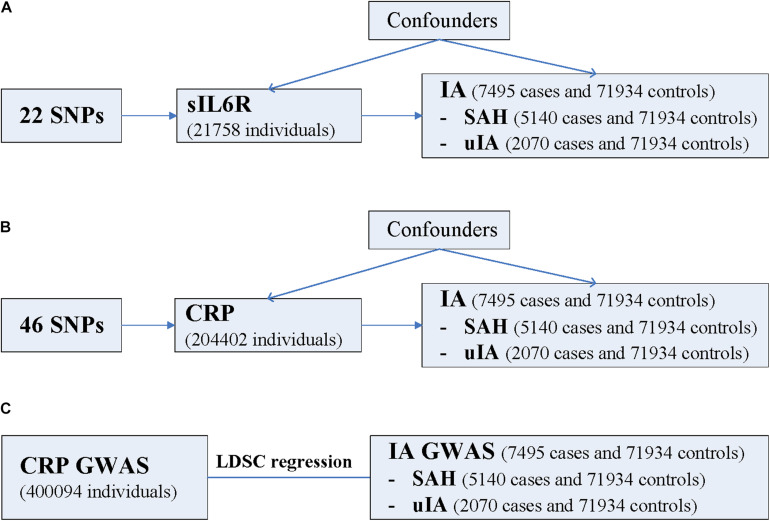
Schematic diagram of the present study. **(A)** MR study of sIL6R levels on risk of IA. **(B)** MR study of CRP levels on risk of IA. **(C)** LDSC regression analysis. CRP, C-reactive protein; GWAS, genome-wide association study; IA, intracranial aneurysm; LDSC, linkage disequilibrium score; MR, Mendelian randomization; SAH, subarachnoid hemorrhage; sIL6R, soluble interleukin-6 receptor; SNP, single nucleotide polymorphism; uIA, unruptured intracranial aneurysm. The three assumptions of the two-sample MR study are as follows: instrument SNPs are associated with exposure; instrument SNPs do not affect the outcome through pathways other than the exposure; and instrument SNPs do not associate with measured or unmeasured confounders. SNPs that meet these three assumptions are considered valid instrument SNPs. Note LDSC regression analysis was not performed for sIL6R because the values of the heritability *z*-score and the mean Chi-square value were too small.

Using summary data of IA from a recently published GWAS (Bakker et al., 2020) as well as GWAS summary data of sIL6R and CRP, we aimed to investigate the causal relationship and genetic overlap between inflammation and IA using the two-sample MR method and LDSC regression (Figure 1).

## Materials and Methods

All analyses are based on publicly available summary data from GWASs. Because ethical approval and informed consent were obtained for each of the original studies, they were not required for the present study.

### Genetic Instruments for sIL6R

Folkersen et al. (2020) conducted a genome-wide meta-analysis of 90 cardiovascular-related proteins in up to 21,758 individuals from 13 cohorts (Table 1). Most of the individuals were of European ancestry. Genetic analyses of the original cohorts were conducted using additive model regressions, adjusting for population structure and study-specific parameters. Individuals with sIL6R levels >3 or 5 standard deviations from the mean were excluded by most original study cohorts. A genome-wide meta-analysis was performed by applying an inverse-variance-weighted approach.

**TABLE 1:** Characteristics of GWAS datasets.

**GWAS data sets**	**Sample size**	**Covariates**	**Notes**
sIL6R	21,758 individuals	Population structure and study-specific parameters.	Individuals with sIL6R levels >3 or 5 standard deviations from the mean were excluded by most original study cohorts.
IA	7,495 cases and 71,934 controls; 5,140 cases and 71,934 controls for SAH subgroup; 2,070 cases and 71,934 controls for uIA subgroup	Sex and 20 principal components. Controls were matched by genotyping platform and country at the cohort level.	Patients with autosomal dominant polycystic kidney disease, Ehlers-Danlos disease, and Marfan’s syndrome were excluded. All controls were unselected controls with no known IA status.
CRP for main MR analyses	204,402 individuals	Age, sex, population substructure, and relatedness.	Individuals with autoimmune diseases, taking immune-modulating agents (if this information was available), or with CRP levels >4 standard deviations from the mean were excluded.
CRP for LDSC regression	400,094 individuals	Age, age^2^, sex, age × sex, age^2^ × sex, and first 20 principal components.	Data was from UK Biobank.

*CRP, C-reactive protein; GWAS, genome-wide association study; IA, intracranial aneurysm; LDSC, linkage disequilibrium score; MR, Mendelian randomization; SAH, subarachnoid hemorrhage; sIL6R, soluble interleukin-6 receptor; uIA, unruptured intracranial aneurysm.*

We selected instruments by selecting all independent SNPs (*r*^2^ < 0.1, in the European 1000 Genome Project reference panel) significantly (*p* < 5 × 10^–8^) associated with sIL6R (UniProt ID: P08887) plasma level for MR analyses (Supplementary Table 1). A total of 85 SNPs were identified. All of these 85 SNPs are located in chromosome 1 with a range of −1836.7 to 410.8 kb around the sIL6R gene (GRCh37 coordinates: 154377819 to 154441926). The F-statistics ranged from 30 to 19,192. *F*-statistics for each SNP was calculated using the following formula: *F* = beta^2^/SE^2^ (Rosa et al., 2019).

### Genetic Instruments for CRP

Ligthart et al. (2018) performed a meta-analysis of GWASs including 204,402 individuals of European ancestry (Table 1). Serum CRP levels were measured using standard laboratory techniques, and the values were transformed by a natural log during analysis. The authors excluded individuals with autoimmune diseases, taking immune-modulating agents (if this information was available), or with CRP levels four standard deviations or more away from the mean. The analysis was corrected for age, sex, population substructure, and relatedness.

The GWAS meta-analyses of CRP revealed 58 distinct genetic loci (*p* < 5 × 10^–8^). A distance criterion of ≥500 kb between two significant variants was used to identify distinct loci. The SNP with the smallest *p*-value at each locus was called the lead variant. We confirmed that the 58 SNPs were independent of each other (*r*^2^ < 0.1, based on the European 1000 Genome Project reference panel) (Supplementary Table 2). The F-statistic ranged from 25 to 2,070.

The CRPGWAS (*n* = 400,094) using the UK Biobank resource is the largest GWAS of CRP. However, a total of 1,243 IA cases and 12,390 IA controls were also from the UK Biobank resources. We did not choose this CRP GWAS to perform the main MR analysis because sample overlap could bias effect estimates from a two-sample MR study (Hemani et al., 2018). However, we performed a sensitivity analysis by using CRP GWAS. The GWAS data are publicly available online^1^ (phenocode: 30710).

### GWAS Summary Data of IA

Summary statistics of IA were obtained from a GWAS of 10,754 cases and 306,882 controls of European and East Asian ancestry (Table 1; Bakker et al., 2020). Patients with autosomal dominant polycystic kidney disease, Ehlers-Danlos disease, and Marfan’s syndrome were excluded. Controls were matched by the genotyping platform and country at the cohort level. All controls were unselected controls with no known IA status. Summary data were accessed through the ISGC Cerebrovascular Disease Knowledge Portal^2^.

Genome-wide association study summary statistics of European ancestry, including 7,495 cases and 71,934 controls, were used in the present MR study. A total of 4,471,083 SNPs passed the quality control thresholds. There were 5,140 SHA cases and 2,070 unruptured IA cases of European ancestry. The proportion of female participants was approximately 55%.

Each of the three summary datasets was used to perform the MR analysis separately. The three summary datasets were as follows: GWAS of IA (ruptured and unruptured) (*n* = 7,495) against controls (*n* = 71,934) in European ancestry individuals, GWAS of SAH-only individuals (*n* = 5,140) against controls (*n* = 71,934) in European ancestry individuals, and GWAS of unruptured IA-only individuals (*n* = 2,070) against controls (*n* = 71,934) in European ancestry individuals.

For instrument SNPs that could not be found in the summary statistics of IA GWAS, a proxy SNP with *r*^2^ ≥ 0.8, was chosen if available.

### MR Analysis

The primary analysis used GWAS summary data of ruptured and unruptured IA together to investigate the causal effect of IL6 signaling and CRP. Secondary analyses were performed using the GWAS summary data of ruptured and unruptured IA separately.

The random-effects inverse variance-weighted (IVW) method was chosen as the main two-sample MR method (Mensah-Kane et al., 2021). The random-effects IVW method allows each SNP to have different mean effects and will return an unbiased estimate if all genetic variants are valid instruments (Figure 1; Bowden et al., 2017). Other MR methods, including MR-Egger, weighted median, weighted mode, simple mode, MR-Mix, MR robust adjusted profile score (MR-RAPS), MR-Robust, and MR-Lasso were chosen to perform sensitivity analyses (Burgess et al., 2019; Qi and Chatterjee, 2019; Slob and Burgess, 2020). These robust analysis methods can provide valid causal inferences under weaker assumptions than the standard IVW method (Burgess et al., 2019; Slob and Burgess, 2020).

Leave-one-out analysis was performed to assess the robustness of the MR estimates by removing each SNP individually. Horizontal pleiotropy was assessed using the intercept of the MR-Egger method. A *p*-value of the intercept less than 0.05 was considered to indicate the presence of horizontal pleiotropy. The MR Pleiotropy RESidual Sum and Outlier (MR-PERESSO) method was used to assess horizontal pleiotropy and to detect potential outlier SNPs (Verbanck et al., 2018). Heterogeneity in IVW and MR-Egger using the Cochran Q statistic was assessed to help to assess horizontal pleiotropy. A *p*-value of Cochran Q statistic less than 0.05 was considered to indicate the presence of heterogeneity.

Previous MR analyses showed that high blood pressure and smoking were causally and independently associated with IA (Bakker et al., 2020). Therefore, sensitivity analyses were performed by excluding instrument SNPs that also associated with blood pressure and smoking at genome-wide significance level (*p* < 5 × 10^–8^) (Liu et al., 2018). GWAS summary data based on UK Biobank resource for blood pressure and smoking from an open GWAS database were used (ID: ukb-b-14177 and ukb-b-10831) (Hemani et al., 2018; Elsworth et al., 2020).

Sensitivity analyses were performed using SNPs within a region of 300 kb window to the sIL6R and CRP genes. We also performed sensitivity analyses using independent (*r*^2^ < 0.1) CRP-associated SNPs (*p* < 5 × 10^–8^) from UK Biobank resources established by Neale et al. (*n* = 400,094) (phenocode: 30710).

A recent MR study used seven SNPs as proxies for IL6 signaling (Georgakis et al., 2020). These seven SNPs were associated with lower CRP levels and within a region of 300 kb window to the sIL6R gene. Three of these were identified in the IA GWAS. Sensitivity analysis was performed using these three SNPs as proxies for IL6 signaling. Estimates and standard errors of these SNPs on CRP levels were used to perform sensitivity analysis.

To validate the MR procedure in the present study, we performed MR analyses using abdominal aortic aneurysm (AAA) and AA as outcomes. These results are consistent with previous MR findings (Supplementary Figure 1; Rosa et al., 2019; Georgakis et al., 2020).

All MR analyses were performed in R (version 3.6.1) using the TwoSampleMR, MendelianRandomization, MR-Mix, MR-RAPS, and MR-PERESSO R packages (Hemani et al., 2018). Since two exposures were chosen for one primary outcome in the MR study and sIL6R was not included in the LDSC regression, a *p*-value of less than 0.017 (0.05/3) was considered statistically significant. A *p*-value less than 0.05, was considered nominally significant.

### Variance Explained by Instrument SNPs

Variance explained for each instrument SNP was calculated using the following formula: 2β^2^MAF(1–MAF) (Park et al., 2010). MAF represents the minor allele frequency of the instrument SNP, and β represents the effect of the effect allele on sIL6R or CRP.

### Statistical Power of MR Analysis

We calculated the statistical power using the tool proposed by Brion et al. (2013). Sample size of IA GWAS, the proportion of cases in the IA GWAS, and variance explained by instrument SNPs jointly were used together to calculate the statistical power under each odds ratio (OR) value.

For primary analysis using sIL6R, there was an 80% power to detect a relative difference of 4.4% (e.g., OR: 1.044/0.956) with an alpha of 5% (Supplementary Table 3). For primary analysis using CRP, there was an 80% power to detect a relative difference of 15.5% (e.g., OR: 1.155/0.845) with an alpha of 5% (Supplementary Table 4).

### LDSC Regression Analysis

We performed cross-trait LDSC regression to estimate the genetic correlations of CRP with IA using GWAS summary statistics. Since LDSC is not biased by sample overlap (Bulik-Sullivan et al., 2015a), we used CRP GWAS (phenocode: 30710) established by Neale et al. (*n* = 400,094) (Table 1) because it includes more individuals than the CRP GWAS by Ligthart et al. (2018) (*n* = 204,402). LDSC was performed using a command line tool (LD score, v1.0.0) (Bulik-Sullivan et al., 2015a,b). LD scores computed using 1000 Genomes European data were used to estimate genetic correlations. Poorly imputed SNPs (imputation INFO < 0.9) were excluded by filtering to HapMap3 SNPs. We excluded SNPs with Chi-square values large than 80 (Rosa et al., 2019), as well as SNPs located in the major histocompatibility complex.

Although the total heritability of sIL6R has been estimated at approximately 70% (Raggi et al., 2010), sIL6R was not included in the LDSC regression because the heritability *z*-score (0.92) was below 4 for SNPs that remain after the filtering. Genetic correlation estimates for traits with small heritability *z*-scores (<4) are generally too noisy to report (Bulik-Sullivan et al., 2015a). Similarly, the mean Chi-square value (1.004) of the SNPs left for LDSC was too small. If the mean Chi-square is too small (e.g., <1.02), the data are probably not suitable for LDSC regression.

The IA GWAS investigated the effects of 376 exposures from the UK Biobank on IA risk (Bakker et al., 2020). Traditional cardiovascular risk factors including high blood pressure, diabetes, hypercholesterolemia, smoking, alcohol drinking, and body mass index were all included. sIL6R and CRP were not involved in the IA GWAS. Because MR analyses showed that high blood pressure and smoking were the only two factors causally and independently associated with IA (Bakker et al., 2020), we used multi-trait conditional and joint analysis to condition IA summary statistics on summary statistics from the UK Biobank GWAS for smoking and blood pressure (Zhu et al., 2018; Bakker et al., 2020). The resulting summary statistics for IA were then used to calculate the genetic correlation between IA and CRP. The European 1000 Genome Project reference panel was used as the reference sample for multi-trait conditional and joint analysis.

## Results

### MR Analyses Based on sIL6R

Among the 85 targeted SNPs, a total of 23 SNPs, including three proxy SNPs were identified in the IA GWAS (Supplementary Table 1). One proxy SNP (rs9427117) was further excluded because it was palindromic with an intermediate allele frequency. The two most significant SNPs were included in the analysis. The variances explained by these two SNPs were 0.43 and 0.07. None of the 22 SNPs were associated with blood pressure (*p* ≥ 0.01) or smoking (*p* ≥ 0.0025) at the genome-wide significance level.

**FIGURE 2:**
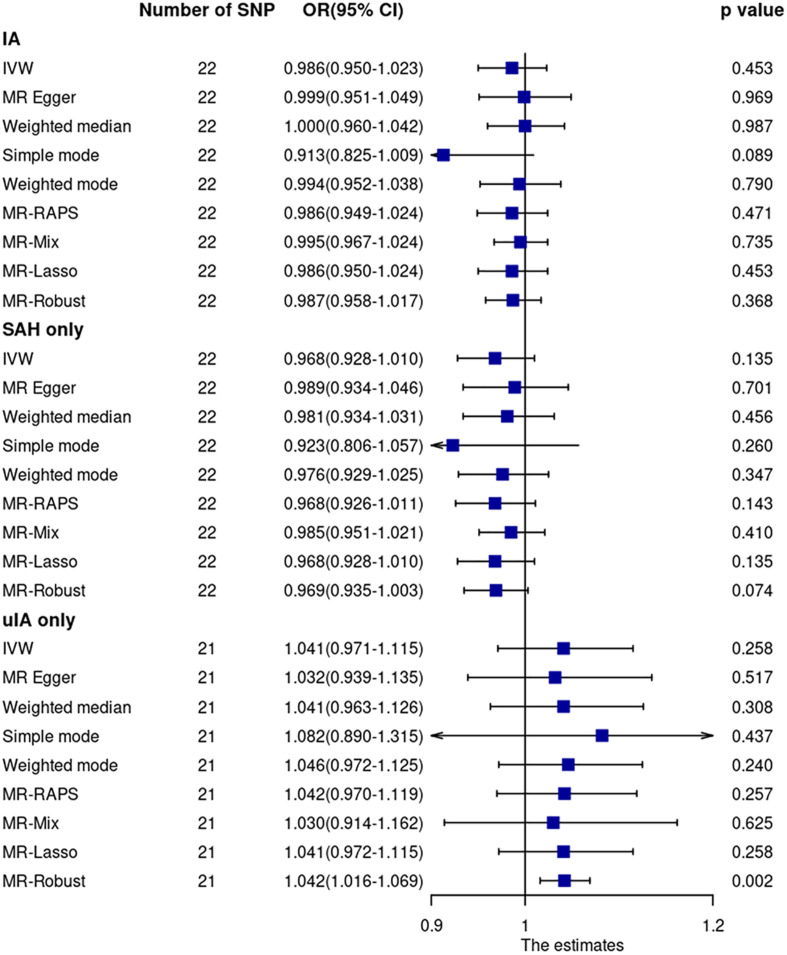
Mendelian randomization analyses of sIL6R levels on risk of IA. CI, confidence interval; IVW, inverse variance weighted; OR, odds ratio; SAH, subarachnoid hemorrhage; SNP, single nucleotide polymorphism; uIA, unruptured intracranial aneurysm.

Mendelian randomization analyses using the 22 SNPs showed that sIL6R was not associated with IA (including ruptured and unruptured IA) (Figure 2 and Supplementary Figure 2). The OR and 95% confidence interval (CI) based on the IVW method were 0.986 (0.950–1.023, *p* = 0.45). The MR analyses using other robust analysis methods showed similar results. Although leave-one-out analysis showed that the estimate was mainly driven by the most significant SNP (rs12126142), the results were not significant after excluding any single SNP (Supplementary Figure 3).

Cochran Q statistic based on IVW (*p* = 0.93) and MR-Egger (*p* = 0.93) showed no evidence of heterogeneity. MR-Egger regression (intercept = –0.0066, SE = 0.0083, *p* = 0.43) (Supplementary Figure 4) and MR-PRESSO global test (*p* = 0.85) showed no evidence of unbalanced pleiotropy. The MR-PRESSO outlier test did not identify outlier SNPs.

Mendelian randomization analysis using GWAS summary data of ruptured and unruptured IA separately showed no evidence of association (Figure 2).

Sensitivity analysis using 15 SNPs within a region of 300 kb window to the sIL6R gene showed no significant association between sIL6R and IA. The OR and 95% CI based on the IVW method were 0.987 and 0.951–1.025 (*p* = 0.50), respectively. Similarly, sensitivity analysis using three CRP-associated SNPs within a region of 300 kb window to the sIL6R gene showed no significant association between IL6-signaling and IA. The OR and 95% CI based on the IVW method were 1.135 and 0.749–1.721, respectively (*p* = 0.55).

### MR Analyses Based on CRP

Among the 58 targeted SNPs, 49 SNPs, including seven proxy SNPs, were identified in the IA GWAS (Supplementary Table 2). Three proxy SNPs were further excluded because they were palindromic with intermediate allele frequencies. The SNP rs2794520, which had the largest *F*-statistic (2,070), was included. The variance explained by this SNP was 0.015.

**FIGURE 3:**
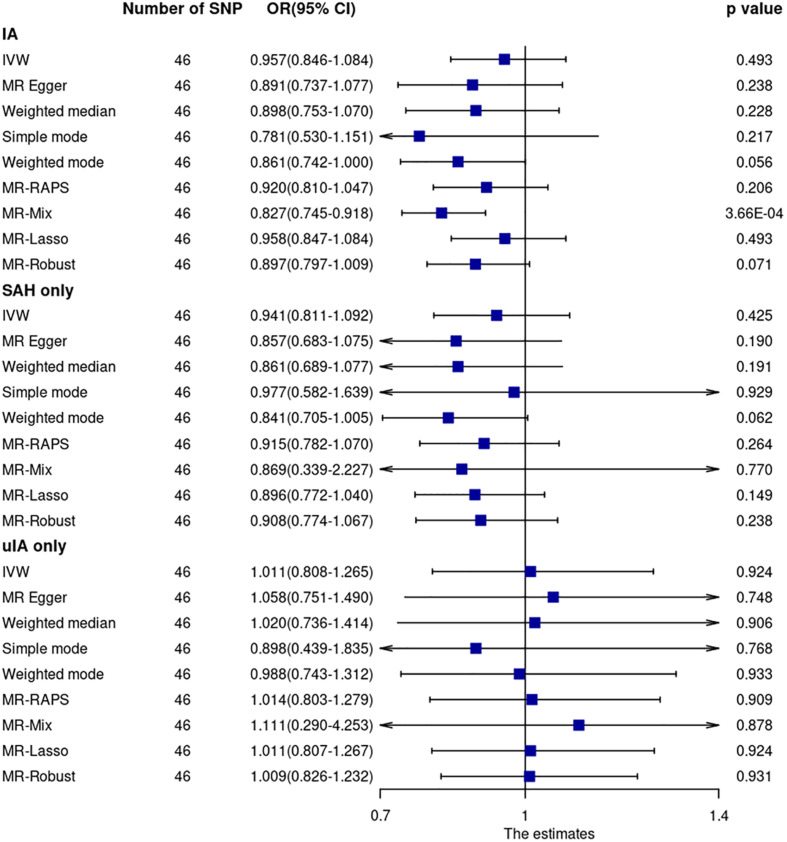
Mendelian randomization analyses of CRP levels on risk of IA. CI, confidence interval; IVW, inverse variance weighted; OR, odds ratio; SAH, subarachnoid hemorrhage; SNP, single nucleotide polymorphism; uIA, unruptured intracranial aneurysm.

Mendelian randomization analyses of the main MR method and most other MR robust methods showed that CRP levels were not associated with IA (including ruptured and unruptured IA) (Figure 3 and Supplementary Figure 5). The OR and 95% CI based on the IVW method was 0.957 (0.846–1.084, *p* = 0.49). However, the MR-Mix method showed that increased CRP levels were significantly associated with decreased IA risk (OR = 0.827, 95% CI = 0.745–0.918, *p* = 3.66 × 10^–4^). Leave-one-out analysis showed that the estimate was not driven by any single SNP (Supplementary Figure 6).

Cochran Q statistic based on IVW (*p* = 0.58) and MR-Egger (*p* = 0.58) showed no evidence of heterogeneity. MR-Egger regression (intercept = –0.0053, SE = 0.00054, *p* = 0.33) (Supplementary Figure 7) and MR-PRESSO global test (*p* = 0.58) showed no evidence of unbalanced pleiotropy. The MR-PRESSO outlier test did not identify significant outlier SNPs.

Mendelian randomization analysis using GWAS summary data of ruptured and unruptured IA separately showed no evidence of association (Figure 3).

None of the 46 SNPs were associated with smoking (*p* > 0.0001) at the genome-wide significance level. Five of the 46 SNPs were associated with blood pressure (rs1558902, rs10838687, rs643434, rs687339, and rs7795281) (*p* < 5 × 10^–8^). Sensitivity analysis by excluding these five SNPs showed similar results (all *p*-values > 0.05). The OR and 95% CI based on the IVW method for IA were 0.947 (0.835–1.075, *p* = 0.40). Sensitivity analysis using CRP GWAS from the UK Biobank resource showed no significant results for all MR methods. The OR and 95% CI based on the IVW method for IA were 0.995 (0.917–1.081, *p* = 0.91). The association between CRP and IA based on the MR-Mix method was dismissed either after excluding the five SNPs associated with blood pressure (OR = 0.861, 95% CI = 0.679–1.091, *p* = 0.22) or after using CRP GWAS from UK Biobank resources (OR = 1.030, 95% CI = 0.634–1.675, *p* = 0.90).

Only one (rs2794520) of the 46 SNPs was within 300 kb of the CRP gene. MR analysis using this SNP showed similar results to those of the main MR analysis.

### LDSC Regression Analysis

There was evidence of a positive genetic correlation between CRP levels and IA. The genetic correlation was relatively small (genetic correlation = 0.16, SE = 0.04, *p* = 0.0003). CRP levels also showed small genetic correlations with SAH and unruptured IA (Supplementary Table 5).

The genetic correlation between CRP levels and IA diminished after conditioning on blood pressure alone (genetic correlation = 0.07, SE = 0.05, *p* = 0.16), smoking alone (genetic correlation = 0.02, SE = 0.05, *p* = 0.65), or blood pressure plus smoking (genetic correlation = −0.03, SE = 0.05, *p* = 0.53).

For comparison, we assessed the genetic correlations of IA with blood pressure and smoking. The genetic correlations were 0.39 ± 0.04 (*p* = 3.61 × 10^–21^) and 0.34 ± 0.05 (*p* = 5.54 × 10^–10^), respectively. These results were consistent with the results of IA GWAS. We further assessed the genetic correlation of IA with blood pressure and smoking, after conditioning on each other. The genetic correlation between IA and blood pressure remained after conditioning on smoking (genetic correlation = 0.33, SE = 0.05, *p* = 4.87 × 10^–13^). The genetic correlation between IA and smoking remained as well after conditioning on blood pressure (genetic correlation = 0.26, SE = 0.06, *p* = 8.06 × 10^–6^).

Finally, we assessed the genetic correlation of CRP levels with blood pressure and smoking. The genetic correlations were 0.31 ± 0.02 (*p* = 3.20 × 10^–45^) and 0.48 ± 0.03 (*p* = 1.93 × 10^–65^), respectively.

## Discussion

Using summary data from GWASs of European ancestry, two-sample MR analyses showed no definite evidence of the causal effects of circulating sIL6R and CRP levels on IA. Although the MR-Mix method showed that increased CRP levels were significantly associated with decreased IA risk, there was no plausible biological explanation for this association. In addition, the association disappeared in the sensitivity analyses. MR analysis using GWAS summary data of ruptured and unruptured IA separately showed no evidence of association. Although LDSC regression showed evidence of a positive genetic correlation between CRP levels and IA, the correlation diminished after conditioning IA on either blood pressure or smoking.

Elevated CRP and IL6 levels are associated with various cardiovascular diseases. Most notably, elevated CRP and IL6 levels have been found to be associated with increased risk of coronary heart disease events in many large prospective observational studies (Casas et al., 2008; Danesh et al., 2008). However, the causality remains uncertain because CRP and IL6 may be mainly markers of established cardiovascular risk factors or markers of subclinical conditions (Casas et al., 2008). An earlier MR study using an IL6R SNP (rs7529229) showed that IL6R signaling had a causal role in the development of coronary heart disease, which supported the causal effect of circulating concentration of IL6 (Interleukin-6 Receptor Mendelian Randomisation Analysis (IL6R MR) Consortium, Swerdlow et al., 2012). Two recent two-sample MR studies using more SNPs further confirmed the causal effect of IL6 signaling on coronary heart disease, as well as the causal effect of IL6 signaling on atrial fibrillation, stroke and AA (Rosa et al., 2019; Georgakis et al., 2020). However, MR studies failed to support the causal association of CRP with many cardiovascular diseases (Elliott et al., 2009; C Reactive Protein Coronary Heart Disease Genetics Collaboration (CCGC), 2011; Georgakis et al., 2020; Wang et al., 2021). These suggested that the effects of IL6 on cardiovascular diseases are independent of the effects of CRP.

Our MR analyses confirmed the causal association of sIL6R with AA, especially AAA (Supplementary Figure 1). Similar to other cardiovascular diseases, this association was independent of the effects of CRP. However, we did not find evidence to support the causal effects of sIL6R and CRP on IA. MR analyses based on the ruptured IA subgroup and unruptured IA subgroup showed no evidence of a causal relationship.

Although the MR method could be used to quantify causal relationships between exposures and outcomes, only SNPs associated with exposures at the genome-wide significance level (e.g., *p* < 5 × 10^–8^) were used. This means the MR method is more suitable for exposures in which the heritability is mainly determined by SNPs at the genome-wide significance level (Bulik-Sullivan et al., 2015a). Unlike the MR method, LDSC regression is more suitable for exposures in which the heritability is mainly determined by thousands of SNPs with small effects (Bulik-Sullivan et al., 2015a). One limitation of LDSC regression is that the LDSC regression itself cannot assess causality. For sIL6R, variance explained by the most significant SNP was as large as 0.434. Conversely, the heritability *z*-score (0.93) and the mean Chi-square value (1.004) after excluding SNPs with large effects (Chi-square > 80) were too small, indicating that the heritability determined by SNPs with small effects was too small to perform LDSC regression. Therefore, the MR method was more suitable than the LDSC regression method for sIL6R. For CRP, the variance explained by the most significant SNP was only 0.015. This means that the MR method may not have enough power to identify the possible small effects of CRP on outcomes such as IA. Moreover, the heritability *z*-score and the mean Chi-square value after excluding SNPs with large effects (Chi-square > 80) were 23.36 and 2.24, indicating that CRP was suitable for LDSC regression.

Because MR analyses showed that high blood pressure and smoking were the only two factors causally and independently associated with IA (Bakker et al., 2020), we assessed the genetic correlation of sIL6R and CRP with IA both before and after conditioning IA on high blood pressure and/or smoking. Although LDSC regression showed evidence of a positive genetic correlation between CRP levels and IA, the correlation diminished after conditioning IA on blood pressure or smoking. In addition, we provided evidence of a positive genetic correlation of CRP with blood pressure and smoking. These suggested that the observed genetic correlation between CRP and IA is mainly driven by the shared genetic background of blood pressure and smoking with CRP and the shared genetic background of blood pressure and smoking with IA.

The present study suggested that aneurysms in different locations respond differently to inflammation caused by IL6 signaling. The difference in response to inflammation can be explained by the extensive differences in pathophysiology and epidemiological risk factors between different aneurysms (Norman and Powell, 2010; van’t Hof et al., 2016). The vessel wall structure differs between the intracranial artery and the aorta. IA usually presents as balloon-shaped or saccular-shaped dilatations, while AA usually presents as fusiform-shaped dilatations. It has been shown that only approximately 7% of patients with AAAs and 5% with thoracic AAs coexist with a cerebral aneurysm, which suggests only a weak association between IA and AAs (Norman and Powell, 2010). A previous study using polygenic analysis and LDSC regression showed no evidence of polygenic overlap between IAs, AAAs, and thoracic AAs (van’t Hof et al., 2016). Although a positive genetic correlation was found between IA and AA in our analyses (Supplementary Table 5), the potential links in the pathophysiology are unlikely including IL6 signaling based on the MR study results in the present study.

There are several limitations to the present study. First, only 22 of the 85 sIL6R-associated SNPs were included in the MR analyses. However, the two most significant SNPs were included. The 22 SNPs had sufficient statistical power to detect a small relative difference in IA risk. Second, the CRP SNPs can only detect a relative difference of 15.5% in IA risk (i.e., OR = 1.155/0.845) with 80% power. Third, although the analyses were performed using GWAS summary data of ruptured (against controls) and unruptured IA (against controls) separately, we did not perform analysis using GWAS summary data of ruptured IA versus unruptured IA. This was because the data set of ruptured IA versus unruptured IA was only available for East Asian ancestry. Furthermore, the sample size for ruptured IA versus unruptured IA (*n* = 3016) was too small to have sufficient statistical power for MR analysis and was too small to perform LDSC regression. Fourth, we only included two markers of inflammation (sIL6R and CRP). Lastly, only GWAS of European ancestry were included, which means that the results may not be generalizable to other populations.

## Conclusion

In conclusion, using summary data from GWASs of European ancestry, two-sample MR analyses showed no evidence of the causal effects of circulating sIL6R and CRP levels on IA risk. MR analysis using GWAS summary data of ruptured and unruptured IA separately showed no evidence of association. Although LDSC regression showed evidence of a positive genetic correlation between CRP and IA, the correlation diminished after conditioning IA on either blood pressure or smoking. The observed genetic correlation between CRP and IA is mainly driven by the shared genetic background of blood pressure and smoking with both CRP and IA.

## Data Availability Statement

The original contributions presented in the study are included in the article/Supplementary Material, further inquiries can be directed to the corresponding author/s.

## Ethics Statement

Ethical review and approval was not required for the study on human participants in accordance with the local legislation and institutional requirements. The Ethics Committee waived the requirement of written informed consent for participation.

## Author Contributions

P-PN and Y-MX: conception and design. P-PN and XW: collection and assembly of data, and data analysis and interpretation. All authors wrote the manuscript and approved the submitted version.

## Conflict of Interest

The authors declare that the research was conducted in the absence of any commercial or financial relationships that could be construed as a potential conflict of interest.

## Footnotes

1 https://pan.ukbb.broadinstitute.org

2 http://www.cerebrovascularportal.org/

## Supplementary Material

Supplementary Figure 1 Forest plots of the estimates of sIL6R and CRP on AA and AAA risk.

Supplementary Figure 2 Forest plot of the estimate of sIL6R on IA risk.

Supplementary Figure 3 Leave-one-out sensitivity analysis of sIL6R on IA risk.

Supplementary Figure 4 Scatter plots of SNP-IL6R and SNP-IA associations.

Supplementary Figure 5 Forest plot of the estimate of CRP on IA risk.

Supplementary Figure 6 Leave-one-out sensitivity analysis of CRP on IA risk.

Supplementary Figure 7 Scatter plots of SNP-CRP and SNP-IA associations.

Supplementary Table 1 Characteristics of sIL6R-associated SNPs.

Supplementary Table 2 Characteristics of CRP-associated SNPs.

Supplementary Table 3 Power to detect a relative difference (in %) in IA risk for a 1-unit difference in log-transformed sIL6R with an alpha of 5%.

Supplementary Table 4 Power to detect a relative difference (in %) in IA risk for a 1-unit difference in log-transformed CRP with an alpha of 5%.

Supplementary Table 5 LDSC regression.

## Abbreviations

AAA: abdominal aortic aneurysm
AA: aortic aneurysm
CI: confidence interval
CRP: C-reactive protein
GWAS: genome-wide association study
IA: intracranial aneurysm
IVW: inverse variance-weighted
LDSC: linkage disequilibrium score
MR: Mendelian randomization
OR: odds ratio
SAH: subarachnoid hemorrhage
sIL6R: soluble interleukin-6 receptor
SNP: single nucleotide polymorphism
uIA: unruptured intracranial aneurysm.
