# Supplementary material for: Association of Interleukin-6 Signaling and C-Reactive Protein With Intracranial Aneurysm: A Mendelian Randomization and Genetic Correlation Study
Source: Front Genet. 2021 Jun 8;12:679363. doi: 10.3389/fgene.2021.679363 (PMC8219052; doi:10.3389/fgene.2021.679363)
Supplement: Supplementary Figure 1 — Forest plots of the estimates of sIL6R and CRP on AA and AAA risk. [file Table_2.DOCX]

**Supplemental Figures**

Figure S1 Forest plots of the estimates of sIL6R and CRP on AA and AAA risk

Figure S2 Forest plot of the estimate of sIL6R on IA risk

Figure S3 Leave-one-out sensitivity analysis of sIL6R on IA risk

Figure S4 Scatter plots of SNP-IL6R and SNP-IA associations

Figure S5 Forest plot of the estimate of CRP on IA risk

Figure S6 Leave-one-out sensitivity analysis of CRP on IA risk

Figure S7 Scatter plots of SNP-CRP and SNP-IA associations

**Figure S1 Forest plots of the estimates of sIL6R and CRP on AA and AAA risk**
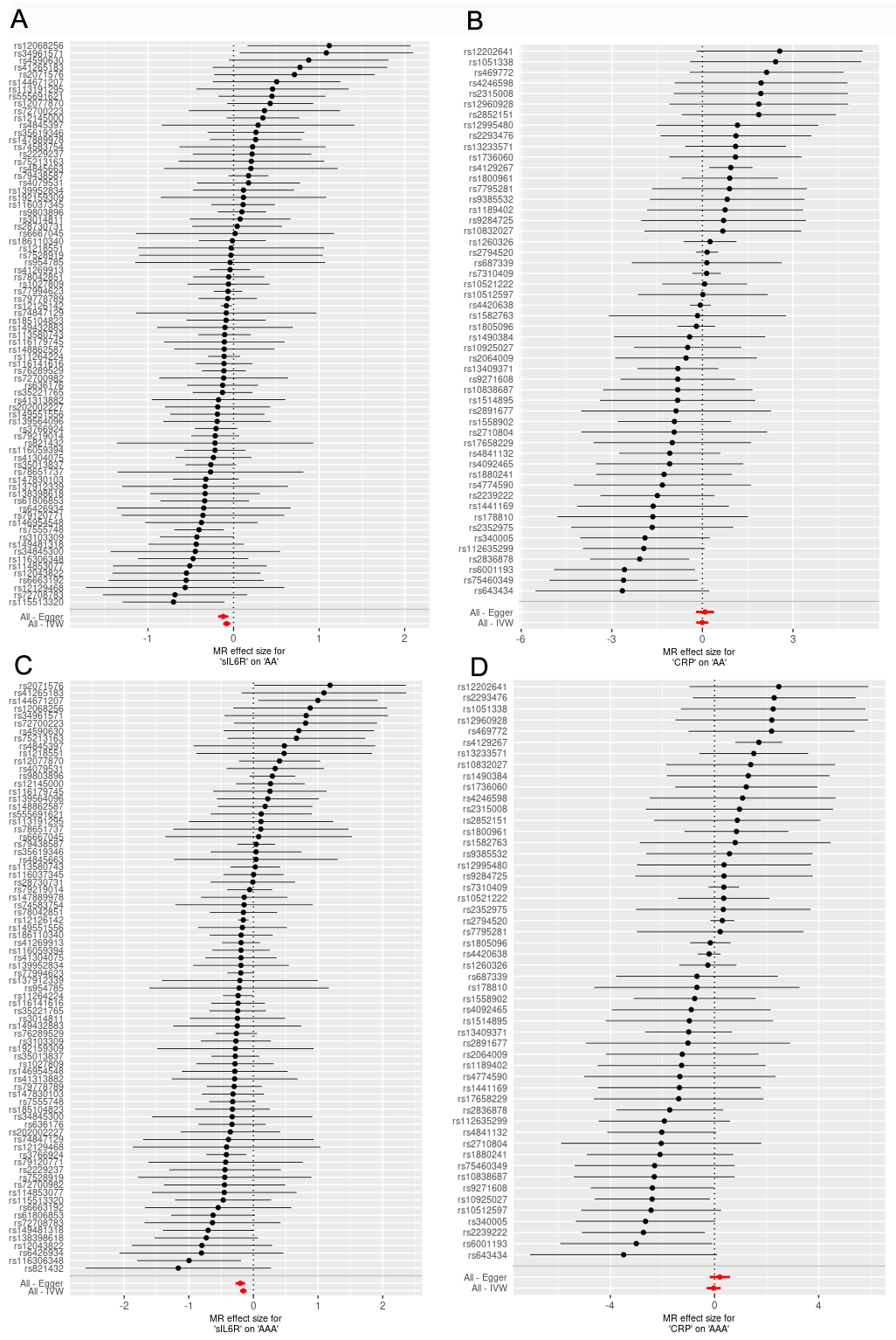


**(A)**: Forest plot of the estimate of sIL6R on AA risk; **(B)**: Forest plot of the estimate of CRP on AA risk; **(C)**: Forest plot of the estimate of sIL6R on AAA risk; **(D)**: Forest plot of the estimate of CRP on AAA risk.

AAA, abdominal aortic aneurysm; AA, aortic aneurysm; CRP, C-reactive protein; IVW, inverse variance-weighted; MR, Mendelian randomization; sIL6R, soluble interleukin-6 receptor.

Summary data of AAA (phenocode: 442.11) and AA (phenocode: 442.1) were established by Neale et al using UK biobank resources. The data are publicly available on a website (https://pan.ukbb.broadinstitute.org). There were 1,306 cases and 2,034 cases of European ancestry for AAA and AA, respectively. There were 408,565 European ancestry controls.

MR analysis confirmed the association between sIL6R and AA. The odds ratio (OR) and 95% confidence interval (CI) based on the IVW method were 0.923 and 0.885–0.962 (p = 1.66×10^−4^).The association was more obvious for AAA (OR = 0.856, 95% CI = 0.813–0.900, p = 1.90×10^−9^). MR analyses showed that CRP was not associated with AA (IVW: OR = 0.998, 95% CI = 0.816–1.221, p = 0.98) or AAA (IVW: OR = 0.968, 95% CI = 0.740–1.268, p = 0.81). These results were consistent with previous MR findings that IL6 signaling was casually associated with AA independent of the effect of CRP.

**Figure S2 Forest plot of the estimate of sIL6R on IA risk**


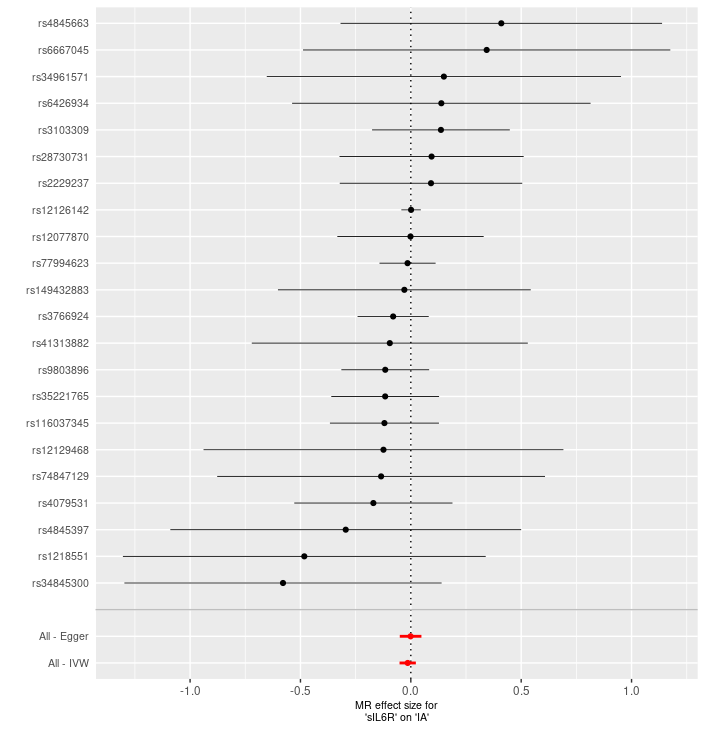


IA, intracranial aneurysm; IVW, inverse variance weighted; MR, Mendelian randomization; sIL6R, soluble interleukin-6 receptor.

**Figure S3 Leave-one-out sensitivity analysis of sIL6R on IA risk**


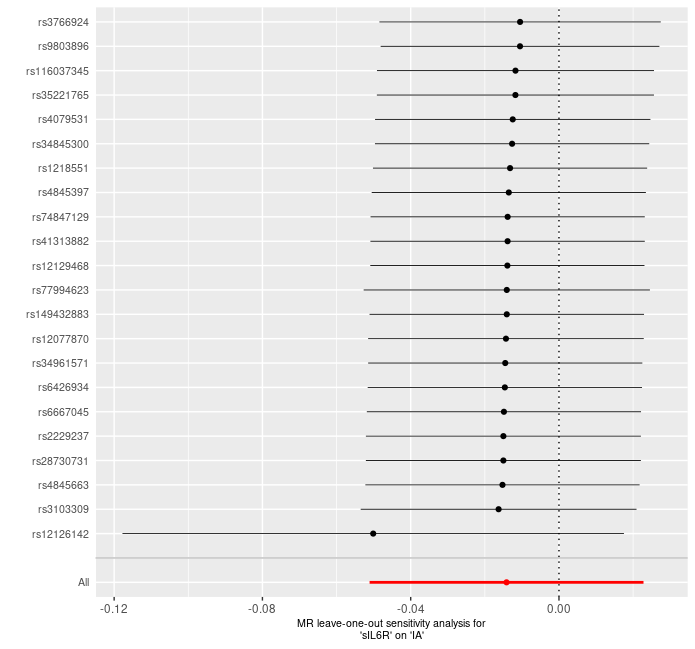


IA, intracranial aneurysm; MR, Mendelian randomization; sIL6R, soluble interleukin-6 receptor.

**Figure S4 Scatter plots of SNP-sIL6R and SNP-IA associations**


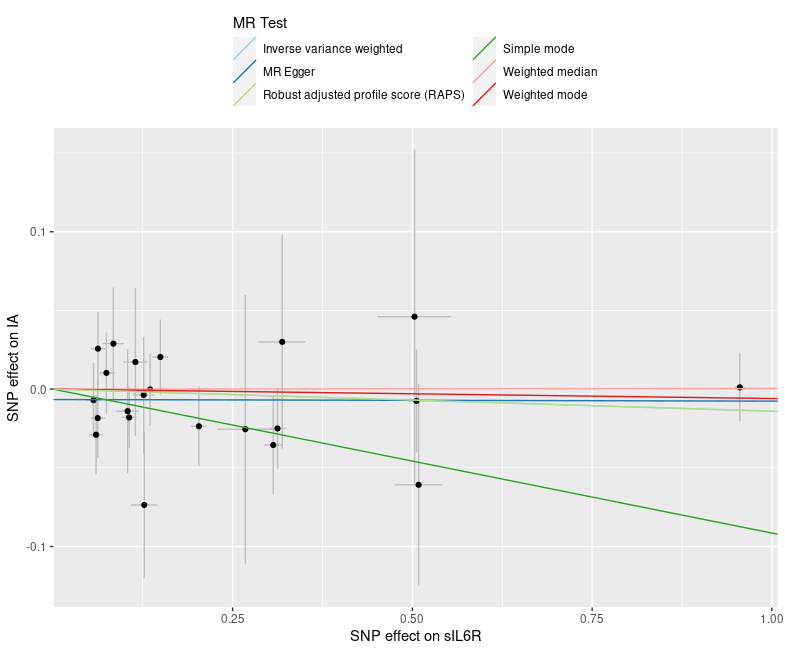


IA, intracranial aneurysm; MR, Mendelian randomization; sIL6R, soluble interleukin-6 receptor; SNP, single nucleotide polymorphism.

**Figure S5 Forest plot of the estimate of CRP on IA risk**


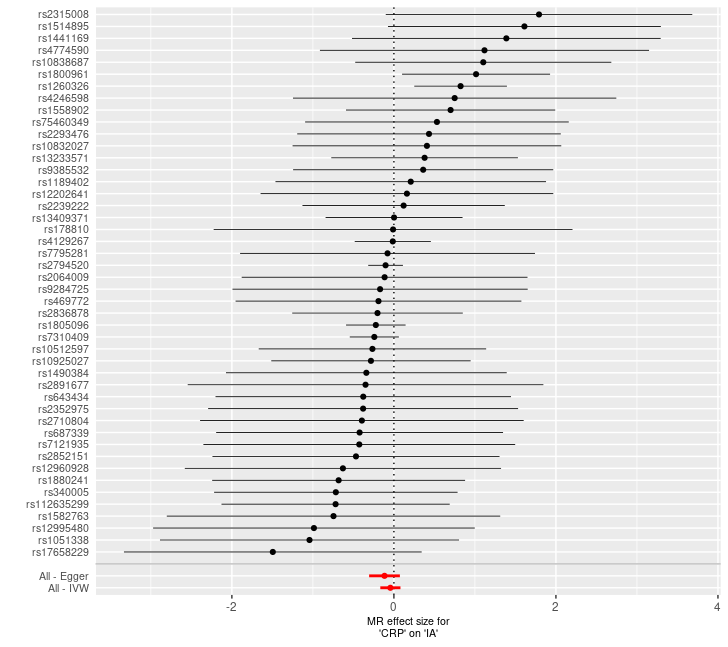


CRP, C-reactive protein; IA, intracranial aneurysm; IVW, inverse variance weighted; MR, Mendelian randomization.

**Figure S6 Leave-one-out sensitivity analysis of CRP on IA risk**


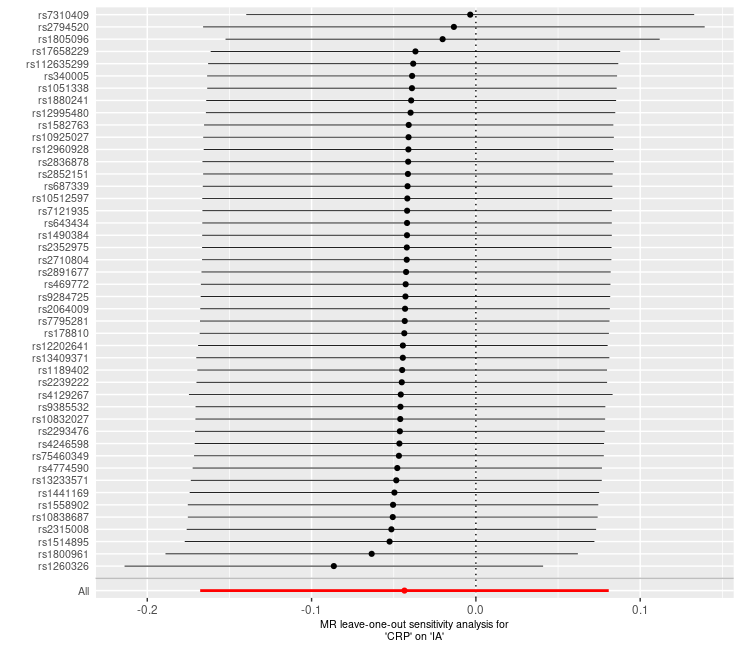


CRP, C-reactive protein; IA, intracranial aneurysm; MR, Mendelian randomization.

**Figure S7 Scatter plots of SNP-CRP and SNP-IA associations**


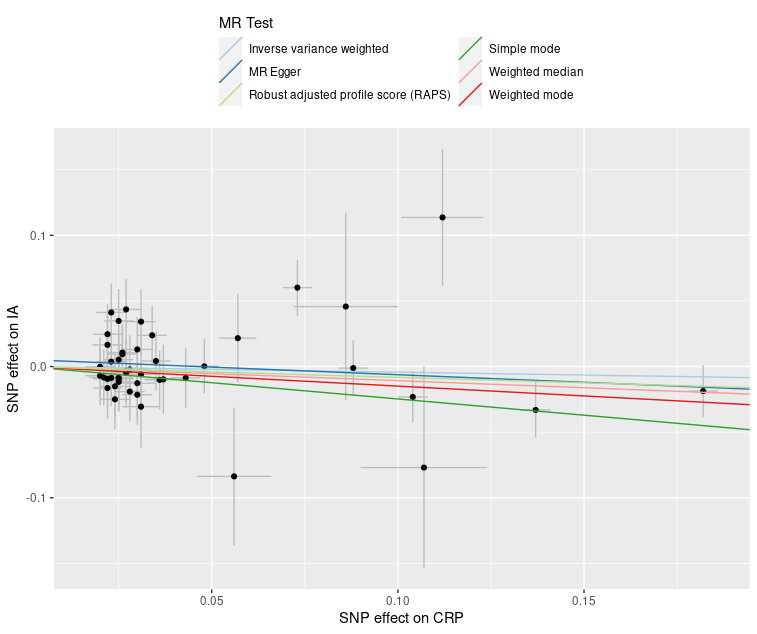


CRP, C-reactive protein; IA, intracranial aneurysm; MR, Mendelian randomization; SNP, single nucleotide polymorphism.
